# Determination of Barbiturates in Biological Specimens by Flat Membrane-Based Liquid-Phase Microextraction and Liquid Chromatography-Mass Spectrometry

**DOI:** 10.3390/molecules24081494

**Published:** 2019-04-16

**Authors:** Ruiqin Zhu, Ying Dong, Xiangyang Cai, Chuixiu Huang

**Affiliations:** Department of Forensic Medicine, Huazhong University of Science and Technology, 13 Hangkong Road, Wuhan 430030, China; zhuruiqin@hust.edu.cn (R.Z.); yingdong@hust.edu.cn (Y.D.); xiangyangc@hust.edu.cn (X.C.)

**Keywords:** membrane-based microextraction, barbiturates, simultaneous determination, whole blood, urine, liver

## Abstract

The wide abuse of barbiturates has aroused extensive public concern. Therefore, the determination of such drugs is becoming essential in therapeutic drug monitoring and forensic science. Herein, a simple, efficient, and inexpensive sample preparation technique, namely, flat membrane-based liquid-phase microextraction (FM-LPME) followed by liquid chromatography-mass spectrometry (LC-MS), was used to determine barbiturates in biological specimens. Factors that may influence the efficiency including organic extraction solvent, pH, and composition of donor and acceptor phases, extraction time, and salt addition to the sample (donor phase) were investigated and optimized. Under the optimized extraction conditions, the linear ranges of the proposed FM-LPME/LC-MS method (with correlation coefficient factors ≥ 0.99) were 7.5–750 ng mL^−1^ for whole blood, 5.0–500 ng mL^−1^ for urine, and 25–2500 ng g^−1^ for liver. Repeatability between 5.0 and 13.7% was obtained and the limit of detection (LOD) values ranged from 1.5 to 3.1 ng mL^−1^, from 0.6 to 3.6 ng mL^−1^, and from 5.2 to 10.0 ng g^−1^ for whole blood, urine, and liver samples, respectively. This method was successfully applied for the analysis of barbiturates in blood and liver from rats treated with these drugs, and excellent sample cleanup was achieved.

## 1. Introduction

Barbiturates, which typically act as central nervous system depressants, are principally used as anxiolytics, hypnotics, and anticonvulsants in medical practice [1,2,3]. Depending on the dosage, barbiturates can produce a wide spectrum of effects [4,5,6]. For example, they can cause relaxation and sleepiness at a relatively low dose but depress the respiratory system at a high dose. Moreover, they have a potential risk of physical and psychological addiction and may result in serious adverse effects [5]. Due to the addictive properties, the use of barbiturates as sedative/hypnotics has largely been superseded by the benzodiazepine group [6]. Nowadays, the wide abuse of such drugs has aroused extensive public concern. Therefore, the determination of barbiturates in biological specimens is not only essential in therapeutic drug monitoring to investigate poisoning but also important in new formulation development, as well as in forensic science [7]. 

In recent decades, the analysis of barbiturates has attracted extensive attention worldwide, and several methods such as ultraviolet–visible spectroscopy (UV-Vis) [8], capillary electrophoresis (CE) [9,10,11,12], liquid chromatography (LC) [13,14], liquid chromatography–mass spectrometry (LC-MS) [15], and gas chromatography–mass spectrometry (GC-MS) [16,17,18,19,20] have been reported for the determination of barbiturates in biological specimens. Traditional technologies such as UV-Vis spectroscopy is still widely used in forensic science for its convenience but it lacks specificity and sensitivity. CE method features high resolving power, low solvent consumption, and simple pretreatment, however, its major drawback is the inherent low concentration sensitivity. GC-MS was commonly applied for the analysis of drugs because it can achieve low limits of detection, however, the sensitivity of GC-MS for barbiturates is not high enough because it requires derivatization. With the recent advances of instruments, LC-MS has become an efficient analytical method to determine barbiturates in drug monitoring due to its high sensitivity and specificity [21]. 

However, it is still difficult to determine the target analyte concentrations at low levels in biological specimens without sample preparation in view of the limited sample volumes and complex sample matrices [8,22,23,24]. Hence, appropriate sample preparation is necessary and of great significance in the whole analysis process. With the development of extraction techniques, miniaturized techniques such as solid-phase microextraction (SPME) and liquid-phase microextraction (LPME) have already been the current trend in sample cleanup [25,26,27,28,29,30,31]. Compared to conventional liquid-liquid extraction (LLE) and solid-phase extraction (SPE), SPME and LPME offer short extraction time and high extraction efficiency without using large volumes of organic solvent. For example, barbiturates in human whole blood, urine, and hair were extracted by SPME and detected by GC-MS [19,32]. However, the construction of a suitable SPME setup often involves a tedious and complicated procedure. Moreover, the fiber used in the SPME suffers from high cost, short life, and the possibility of carryover [33].

On the other hand, LPME, which is considered as a “green” extraction technique, has attracted widespread attention for its good purification capability, economical efficiency, and easy operation [18,28,29,30,31,34]. Until now, LPME has already been applied in the purification and enrichment steps in different biological samples [8,16,18,23,34]. For example, Zarei et al. adopted a dispersive liquid–liquid microextraction (DLLME) technique combined with spectrophotometric analysis for the determination of trace amounts of barbituric acid in human serum [8]. Hollow fiber–liquid phase microextraction (HF-LPME) was also developed to isolate barbiturates in hair [18], blood [16], and liver samples [35], and coupled with GC-MS, satisfactory results can be reached.

**Figure 1:**
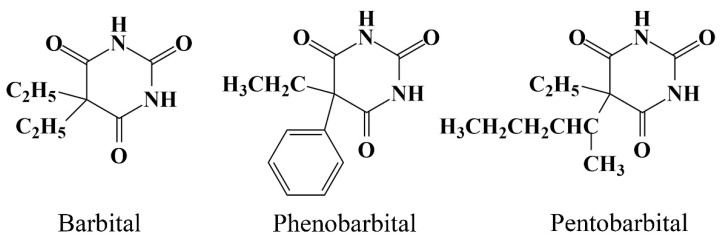
Structure diagram of barbital, phenobarbital, and pentobarbital.

Taking advantage of LPME, in this work, we aim to develop a method for the simultaneous quantification of barbital, phenobarbital, and pentobarbital in urine, blood, and liver tissue (the structures of the three barbiturates can be found in Figure 1). In our previous work [29], flat membrane-based liquid-phase microextraction (FM-LPME) was applied for the extraction of acidic drugs from human plasma. Compared to hollow fibers, the flat membrane device can accommodate a larger amount of acceptor phase to promote high efficiency. In addition, the FM-LPME setup is more convenient and easier to manipulate. Here, LC-MS, which has a lower limit of detection and no requirement for pretreatment of derivatization compared to GC-MS, is applied to detect the target analytes. To the best of our knowledge, this is the first report on the analysis of barbiturates in biological samples using FM-LPME coupled with LC-MS.

## 2. Results and Discussion

### 2.1. Optimization of the Extraction Conditions

In order to optimize LPME of barbiturates from biological specimens, we systematically studied the analytical factors such as solvent type, donor and acceptor phase type, extraction time, and salt addition that may potentially affect the sample extraction efficiency. In this study, the extraction efficiency is defined as extraction recovery, and the recovery for each analyte was calculated by the following equation:

$$\mathrm{Recovery}=C_{A}\times \frac{V_{A}}{C_{D}^{0}\times V_{D}}\times 100\%\;$$

where $C_{A}$ represents the concentration of the analytes in the acceptor solution after extraction, and $C_{D}^{0}$ is the initial concentration of the analytes in the sample solution, while $V_{A}$ and $V_{D}$ are the volumes of the acceptor and sample solution, respectively.

#### 2.1.1. Selection of the Organic Extraction Solvent

**Figure 2:**
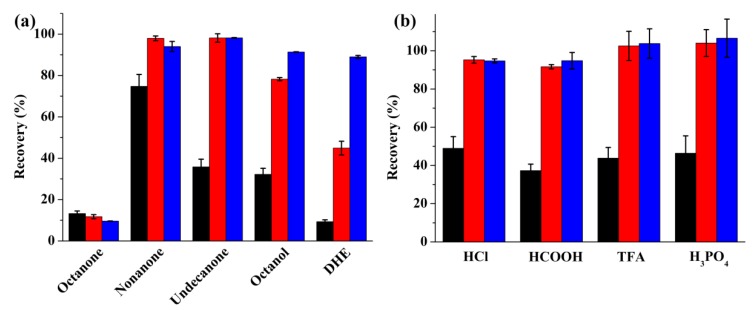
Extraction efficiency for barbiturates (black, red, and blue columns represent barbital, phenobarbital, and pentobarbital, respectively) using different organic solvents (**a**) and donor phases (**b**). Extraction conditions: extraction time: 60 min; stirring speed: 1000 rpm; acceptor phase: 100 μL 20 mM Na_3_PO_4_; (**a**) donor phase: 800 μL 10 mM HCl and 4 μL different organic solvent; (**b**) organic solvent: 4 μL 2-nonanone and 800 μL donor phase.

The type of organic solvent is a key parameter in LPME. The organic solvent is impregnated in the pores of the membranes, constructing the supported liquid membrane (SLM). Therefore, the ideal organic solvent should be compatible with the membrane, immiscible in the donor and acceptor phase, and have good stability over the extraction process and excellent affinity for the target analyte [36]. On the basis of these considerations, we tested five types of organic solvents including 2-octanone, 2-nonanone, 2-undecanone, 1-octanol, and dihexyl ether (DHE). As shown in Figure 2a, 2-nonanone provided the highest extraction recovery for barbiturates among the tested organic solvents. Thus, 2-nonanone was selected and used in the rest of the experiments.

#### 2.1.2. Optimization of the Donor Phase

Barbiturates are acidic drugs with a pKa at about 7. In order to get high extraction efficiency, the donor phase should be acidified so that the analytes can be deionized and consequently transfer from the donor phase into the organic phase. In this study, four different acids including hydrochloric acid, formic acid, trifluoroacetic acid (TFA), and phosphoric acid were tested at a concentration of 10 mM. The results in Figure 2b display no obvious difference using the four acids. The pH-value of all the above-selected donor phase background electrolytes are below 3, meaning that the targets can be completely deionized in all of the tested donor phases. Therefore, recoveries of all three barbiturates were similar to the tested donor phases. Here, hydrochloric acid was selected for the subsequent experiments because it provided the highest recovery for barbital and it is one of the most commonly used background electrolytes in LPME.

#### 2.1.3. Optimization of the Acceptor Phase

**Figure 3:**
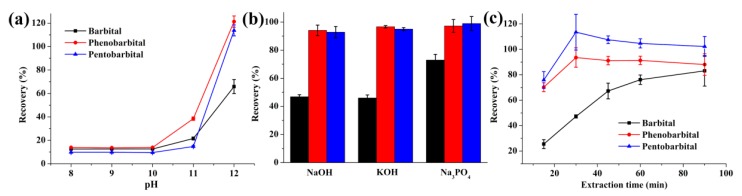
Effect of pH (**a**), composition of acceptor phase (**b**), extraction time (**c**) on the recovery of barbiturates. In (**b**), the black, red, and blue columns represent barbital, phenobarbital, and pentobarbital, respectively. Extraction conditions: stirring speed: 1000 rpm; donor phase: 800 μL 10 mM HCl; 4 μL 2-nonanone (**a**) 100 μL acceptor phase with different pH and 60 min extraction time; (**b**) 100 μL different acceptor phases with pH 12 and 60 min extraction time (**c**) 100 μL trisodium phosphate with pH 12 and different extraction time.

Since barbiturates are acidic analytes, the acceptor phase should be basic to ionize them in order to prevent the analytes from re-entering into the organic phase [37]. In this study, the pH of the acceptor phase was adjusted in the range of 8–12 using sodium hydroxide. According to the results in Figure 3a, the highest recovery was obtained at a pH value of 12, and higher pH (≥ 13) resulted in an M-shaped peak for barbital and phenobarbital. As a result, further studies were conducted with an acceptor solution pH 12.

For the determination of the composition of the acceptor phase, three different basic chemicals including sodium hydroxide, potassium hydroxide, and trisodium phosphate were tested. As observed in Figure 3b, trisodium phosphate gave the best recovery for barbiturates. Trisodium sodium as the acceptor solution obtained the best extraction efficiency, possibly due to its better buffering capacity, thus, facilitating pH gradient transport of the targets.

#### 2.1.4. Effect of Stirring Rate and Extraction Time

Normally, stirring of the sample solution can accelerate the extraction because it facilitates the analyte diffusion from the donor phase to the interface of the SLM [37], reducing the time required to reach thermodynamic equilibrium. The effect of sample agitation was tested using a stirring speed between 250 and 1000 rpm. It was observed that with an increase in the stirring rate, the barbiturate extraction efficiencies were improved. Therefore, 1000 rpm was chosen as the optimal stirring speed in subsequent experiments.

The mass transfer kinetics of LPME is passive diffusion, so it takes time for the analytes to reach equilibrium within the three phases. As a consequence, the extraction time can influence the distribution of the analyte between the sample, SLM, and acceptor phase. Therefore, the influence of extraction time (from 15 to 90 min) on the recovery of barbiturates was investigated (Figure 3c). It was clearly shown that with an increase in the extraction time up to 30 min, the recovery increased rapidly for all three barbiturates but decreased slightly thereafter for pentobarbital and phenobarbital because the system reached equilibrium. For barbital, the recovery kept increasing with extending the extraction time. It has been reported that the validation data are not affected by the extraction time under non-equilibrium conditions using LPME [38]. From the view of practical application, we selected 60 min as the extraction time for the following experiments. 

### 2.2. FM-LPME of Barbiturates from Biological Specimens

**Figure 4:**
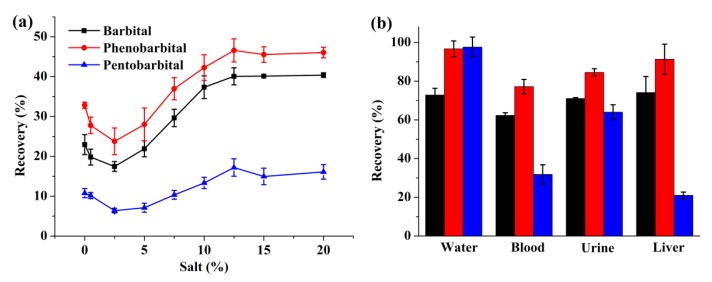
(**a**) The effect of salt addition on the extraction of barbiturates from the whole blood sample; and (**b**) extraction efficiencies of barbiturates from different samples applying FM-LPME. Black, red, and blue columns represent barbital, phenobarbital, and pentobarbital, respectively. Extraction conditions: extraction time: 60 min; stirring speed: 1000 rpm; acceptor phase: 100 μL 20 mM Na_3_PO_4_; organic solvent: 4 μL 2-nonanone.

Subsequently, the optimized extraction procedures were performed on biological specimens including whole blood, urine, and liver. The addition of salt in the blood sample may increase the recovery in microextraction procedures, especially for the more polar analytes because of the salting-out effect [38]. Moreover, the diffusion of analytes might be reduced due to the interaction of the analyte molecules with the added ions [39]. For that purpose, the salt influence was tested with the addition of NaCl at concentrations between 0.5 and 20% (*w*/*v*) in the extraction solution in whole blood samples. As shown in Figure 4a, with the addition of salt, the extraction efficiency decreased initially and then increased steadily until the salt concentration reached 12.5%. As a result, a concentration of 12.5% of salt was added to the whole blood samples to improve the extraction efficiency. Due to the viscosity of the biological samples, appropriate dilution has an apparent effect on recovery improvement. As a result, the whole blood, urine, and homogenized liver were diluted with HCl solution by different times before the extraction procedure, which is depicted in detail in the Materials and Method part. The extraction efficiencies for the biological samples and water sample are shown in Figure 4b. Compared with the water sample, lower recoveries were obtained from the biological samples for all three barbiturates. It has already been proven that barbiturates tend to bind to proteins in biological samples, and protein adsorption to the membrane surface also leads to a lower mass transport rate during the extraction [9]. A distinct decrease could be observed in the recovery of pentobarbital from the blood and liver samples, which might have resulted from the stronger protein binding ability of pentobarbital [40]. Premised on these considerations, the extraction efficiencies from the biological samples could be regarded as satisfying.

### 2.3. Method Evaluation

To evaluate the analytical performance of the proposed method, figures of merit of this method including linear range, limit of detection and quantification (LOD and LOQ), and repeatability were studied for the extraction of barbiturates from biological samples under the optimum conditions and the results are illustrated in Table 1.

The calibration curves show good linearity for all analytes in the ranges as shown in Table 1 with correlation coefficient factors all greater than 0.99. The LODs for whole blood were 1.5–3.1 ng mL^−1^, for urine were 0.6–3.6 ng mL^−1^, and for liver were 5.2–10.0 ng g^−1^. It was reported that the therapeutic blood levels of the barbiturates were several µg ml^−1^ to several 10 µg mL^−1^ [41]. Therefore, the proposed method is sensitive enough to meet the therapeutic levels. 

The repeatability of the proposed method was evaluated by analyzing the biological specimens spiked with barbiturates (*n* = 5) at a concentration of 50 ng mL^−1^. Repeatability results are expressed as relative standard deviation (RSD %). The RSD values were below 20% in all cases. The assay results demonstrate that our present method can provide good repeatability for complex biological specimens. 

### 2.4. Application

Due to the importance of determining barbiturates in biological specimens, the optimized and evaluated method was applied to determine the concentration of barbiturates in whole blood and liver samples from two rats treated with barbiturates. Two male Sprague-Dawley (SD) rats weighing 250 g and 300 g were gavaged with a barbiturate mixture at a dose of 50 mg kg^−1^ [42]. Then, two hours later, their whole blood and liver were collected for analysis, and the results are summarized in Table 2. 

### 2.5. Comparison of the Proposed Method with Other Reported Methods

Table 3 summarizes different methods reported in the literature for determining barbital, phenobarbital, and pentobarbital in biological samples. As can be observed from the table, compared with other reported methods, our method provided almost the lowest LOD value for all three barbiturates.

## 3. Materials and Methods 

### 3.1. Chemicals and Materials

Barbital was obtained from Shenyang Trial Three Biochemical Technology Development Co., Ltd. (Shenyang, China). Phenobarbital and pentobarbital sodium were purchased from Shanghai Chemical Reagent Factory (Shanghai, China). Formic acid, acetic acid, 2-octanone, 2-nonanone, 2-undecanone, 1-octanol, dihexyl ether (DHE), trifluoroacetic acid (TFA), and diclofenac sodium were all purchased from Aladdin Chemical Reagent Co. (Shanghai, China). Hydrochloric acid (HCl), sodium hydroxide (NaOH), potassium hydroxide (KOH), and trisodium phosphate (Na_3_PO_4_) were supplied by Sinopharm Chemical Reagent Co., Ltd. (Shanghai, China). Methanol was from Tedia (Fairfield, OH, USA). Formic acid, methanol, and acetic acid were of chromatographic purity grade while other chemicals were of analytical grade. Milli-Q water purification system (Mollsheim, France) was employed to produce deionized water.

Accurel PP 1E (R/P) flat membrane (polypropylene membrane, average thickness of 100 μm) was from Membrana (Wuppertal, Germany). The standard 1000 μL pipette tips were from Kirgen (Shanghai, China). The Eppendorf safe lock 2.0 mL PP tubes were obtained from Eppendorf AG (Hamburg, Germany).

### 3.2. FM-LPME Setup and Extraction Procedures

**Figure 5:**
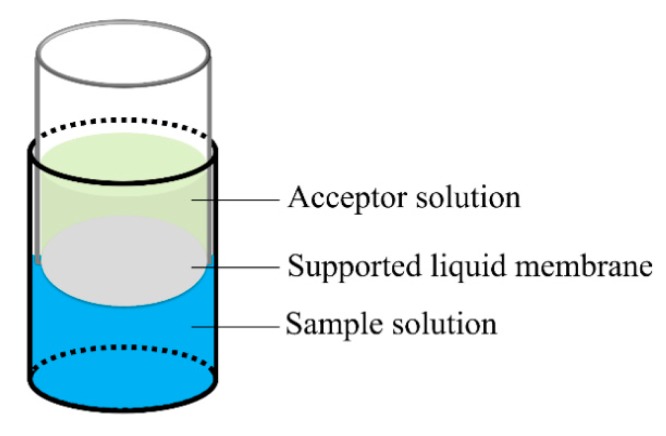
Schematic representation of flat membrane-based liquid-phase microextraction (FM-LPME) setup.

The setup diagram of FM-LPME is shown in Figure 5. It comprised two aqueous phases―acceptor phase and donor phase―isolated by SLM, which was prepared by immobilization of some organic solvent into the pores of the Accurel PP 1E (R/P) flat membrane. The fabrication of this FM-LPME configuration was described in our previous work [44]. The container of acceptor solution was a wide end-closed 1000 μL pipet tip sealed with a piece of flat membrane. The narrow end of the pipet tip was cut off for easy operation. The donor compartment was a 2 mL Eppendorf PP tube. Afterward, the acceptor compartment was inserted into the sample compartment. The LPME process was initiated by starting the MIC-100 constant temperature mixer (Hangzhou MIULAB Instrument Co. Ltd., Hangzhou, China) with a speed of 1000 rpm. After the default extraction time, the extraction was terminated by manually turning off the agitator. Immediately, the acceptor solution after LPME was collected individually and subsequently analyzed by HPLC-UV or LC-MS.

### 3.3. Sample Preparation 

#### 3.3.1. Water Samples 

The stock solution of barbiturates was prepared by dissolving the drugs in methanol with a concentration of 1 mg mL^−1^ and stored at 4 °C in the dark. The working solution (water sample) was obtained by diluting the stock solutions to a concentration of 5 µg mL^−1^ with 10 mM HCl solution. 

#### 3.3.2. Biological Samples

Drug-free whole blood, urine, and liver were used for the optimization of the LPME conditions and the validation of the proposed analytical method. For the validation of FM-LPME-LC-MS, diclofenac sodium (50 ng mL^−1^) was used as the internal standard (IS). The drug-free whole blood and urine samples were collected from the volunteers who had not been exposed to barbiturates. Liver specimens were obtained from Tongji Medicolegal Expertise Center in Hubei.

The whole blood sample was diluted three times using 10 mM HCl solution containing NaCl, three barbiturates at desired concentration, and IS (if applicable). The urine sample was diluted 1:1 with 10 mM HCl containing the three barbiturates at the desired concentration and IS (if applicable). 

One gram liver tissue was weighed and homogenized with Freezer Mixer (Shanghai Jingxin Industrial Development Co. Ltd., China) after overnight lyophilization. The homogenization of lyophilized liver tissue was conducted for 30 min in a 5 mL grinding jar containing 3 mL of 10 mM HCl and four grinding beads at 60 rpm. The homogenized sample was transferred to a 5 mL volumetric flask and diluted to 5 mL (with the liver concentration of 0.2 g mL^−1^) using 10 mM HCl. Prior to FM-LPME, the mixture was 1:1 (*v*/*v*) diluted with 10 mM HCl containing barbiturates and IS (if applicable) and equilibrated overnight at 4 °C in the dark.

#### 3.3.3. Method Evaluation

In order to assess the practical applicability of the proposed method, extractions were performed under the optimal conditions for diluted whole blood, urine, and liver tissue spiked with model analytes at concentrations of 1, 2, 5, 10, 20, 50, 100, 200, and 500 ng mL^−1^ (*n* = 4 for each concentration). Moreover, diclofenac sodium with a concentration of 50 ng mL^−1^ was used as the internal standard (IS). The acceptor solution after FM-LPME was then analyzed by LC-MS. The peak area ratio between analyte and IS versus analyte concentration was plotted to construct the calibration curve. LOD and LOQ were determined to estimate the sensitivity of the method and calculated as the concentration of the inject sample to yield a signal-to-noise ratio of three and ten, respectively.

To assess repeatability of the method, replicate analysis (*n* = 5) of biological samples spiked at ng mL^−1^ of each analyte was performed. Repeatability results are expressed as RSD from the replicate theoretical value. 

#### 3.3.4. Application Experiment

Two male SD rats weighing 250 g and 300 g were first fed with a mixture of the three barbiturates at a dose of 50 mg kg^−1^ by gavage. Two hours later, the whole blood and liver tissue of the rats were collected individually by open chest cardiac puncture and laparotomy, respectively. The whole blood was diluted 100 times with 10 mM HCl solution containing 12.5% NaCl and 50 ng mL^−1^ of IS was used as the donor phase of FM-LPME. The liver samples for FM-LPME were prepared as described above. The concentration of the barbiturates in the liver was out of the linearity range for FM-LPME/LC-MS. Therefore, the liver samples for FM-LPME were finally diluted with 10 mM HCl to a concentration of approximately 2.5 mg mL^−1^. The diluted liver samples were mixed 1:1 with 10 mM HCl solution containing IS and equilibrated overnight at 4 °C in the dark before FM-LPME. The acceptor phases were collected and analyzed by LC-MS. All procedures related to animals were in accordance with the international, national, and/or institutional guidelines and were approved by the Institutional Animal Care and Use Committee of Tongji Medical College of Huazhong University of Science and Technology in January 10, 2018 ([2018] IACUC Number: 2127).

### 3.4. HPLC-UV Analysis

An Ultimate 3000 system equipped with a pump (LPG-3400RS), an autosampler (WPS-3000RS), a column oven (TCC-3000RC), and a VWD-3400RS UV/Vis detector (all from Thermo Scientific, Waltham, MA, USA) was used for the chromatographic separation, and the UV-Vis detector was operated at 214 nm. Data were collected and processed by Chromeleon software 7.2 SR5 (Thermo Scientific, Waltham, MA, USA). Separation was carried out on a Hypersil GOLD C18 column (100 mm × 2.1 mm, 5 μm) (Thermo Scientific, Waltham, MA, USA) at 45 °C, with an injection volume of 10 μL. Mobile phase A was 20 mM formic acid containing 5% of methanol (*v*/*v*), and mobile phase B was methanol containing 5% of 20 mM formic acid (*v*/*v*). Mobile phase B was increased from 20% to 80% within 3.5 min at a flow rate of 0.8 mL min^−1^; afterward, it was decreased to 20% within 0.1 min, and this condition was kept for 2.5 min for equilibration.

### 3.5. LC-MS Analysis

Analysis of barbiturates was performed using an Ultimate 3000 UPLC system interfaced with a TSQ Quantum Access MAX triple quadrupole Mass Spectrometry (Thermo Scientific, Waltham, MA, USA). Chromeleon client software (Thermo Scientific, Waltham, MA, USA) was used for LC control, and Xcalibur software (Thermo Scientific, Waltham, MA, USA) was used to control the MS, data acquisition, and data processing. Chromatographic separation was conducted at 45 °C on an Accucore C18 column (2.1 mm × 100 mm, 2.6 μm) (Thermo Scientific, Waltham, MA, USA) with gradient elution. Water containing 0.5% of acetic acid (*v*/*v*) was used as mobile phase A, and methanol was used as mobile phase B. Mobile phase B started from 20% for 0.5 min, and then increased to 95% in 1.5 min and 95% was kept for 2 min. At the end, mobile phase B was decreased to 20% within 0.1 min, which was kept for 1.9 min for equilibration. The flow rate was set to 0.4 mL min^−1^ and the injection volume was 10 μL. Mass spectrometry was performed with an ESI source in the negative-ionization mode with a sheath gas of 40 Arb and aux gas of 10 Arb. The capillary temperature was set at 320 °C and the vaporizer temperature was set at 350 °C. The spray voltage was 3.2 kV. The parameters for the quantification selected reaction monitoring (SRM) transitions are presented in Table 4.

## 4. Conclusions

In this study, a simple, efficient, and inexpensive sample preparation technique, namely, FM-LPME coupled with LC-MS, was used to determine barbiturates in biological specimens. Compared to hollow fibers, the flat membrane device is easier for operation and can house a larger amount of acceptor solution, which might lead to high recovery. To our best knowledge, this is the first report on the simultaneous measurement of three barbiturate drugs in human biological specimens using FM-LPME/LC-MS analysis. Three barbiturate drugs could be rapidly and efficiently extracted and simultaneously determined even at trace concentration. This established method has the potential to be used in therapeutic drug monitoring and clinical toxicology, as well as forensic toxicology. 

## Figures and Tables

**Table 1:** Validation of the proposed method for determination of the three barbiturate drugs in biological specimens.

Matrices	Analytes	Linearity (ng mL^−1^)	LOD (ng mL^−1^)	LOQ (ng mL^−1^)	Repeatability (%)
Blood	Barbital	15–750	2.3	7.7	10
	Phenobarbital	7.5–750	1.5	5.0	6
	Pentobarbital	15–750	3.1	10.2	8
Urine	Barbital	20–500	3.6	12.0	11
	Phenobarbital	5–500	1.2	4.0	5
	Pentobarbital	5–500	0.6	2.0	5
Liver ^1^	Barbital	50–2500	10.0	33.3	9
	Phenobarbital	25–2500	5.2	17.3	11
	Pentobarbital	25–2500	7.4	24.7	14

^1^ The concentration unit for liver is ng g^−1^.

**Table 2:** Concentrations of barbiturates in whole blood and liver found in two actual cases.

	Analytes	Blood	Liver
		Drug Concentration ^1^ (µg mL^−^^1^)	Drug Concentration ^1^ (µg g^−^^1^)
Rat 1	Barbital	51.1 ± 4.9	17.2 ± 3.0
	Phenobarbital	50.3 ± 4.1	18.8 ± 3.2
	Pentobarbital	36.0 ± 2.4	52.6 ± 8.2
Rat 2	Barbital	44.9 ± 3.0	19.6 ± 1.5
	Phenobarbital	44.8 ± 6.7	20.0 ± 1.6
	Pentobarbital	34.2 ± 3.7	58.9 ± 4.4

^1^ The concentrations were calculated based on the dilution times.

**Table 3:** Summary of reported methods for determining barbiturates in biological specimens.

Sample	Analytes	Extraction	Detection	Linear Range (ng mL^−1^)	LOD (ng mL^−1^)	Ref
Urine	Phenobarbital Barbital	SPE	CE	2–500	0.5–5.0	[11]
Blood	Pentobarbital	SPME	GC-MS	200–40000	50	[32]
Serum	Barbital Phenobarbital	LLE	CE-UV	2900–43290	830–1390	[12]
Liver	Pentobarbital Phenobarbital	HF-LPME	GC-MS	1000–10,000 (ng g^−1^)	500 (ng g^−1^)	[35]
Blood	Pentobarbital Phenobarbital	HF-LPME	GC-MS	1000–10,000	1000	[16]
Blood	Barbital Phenobarbital Pentobarbital	LLE	LC-MS	2–2000	0.2–0.5	[43]
Blood Urine Liver	Barbital Phenobarbital Pentobarbital	FM-LPME	LC-MS	7.5–750 ^1^ 5–500 ^2^ 25–2500 ^3^ (ng g^−1^)	1.5–3.1 ^1^ 0.6–3.6 ^2^ 5.2–10.0 ^3^ (ng g^−1^)	Our work

^1^ For blood. ^2^ For urine. ^3^ For liver.

**Table 4:** Mass spectrometry parameters for three barbiturates and internal standard (IS).

Analyte	Parent (*m*/*z*)	Product (*m*/*z*)	Collision energy (eV)	Tube lens (V)	Retention Time (min)
Barbital	183.0	42.4	80	52	1.54
		140.0	13		
Phenobarbital	231.0	42.4	17	57	3.33
		188.0	10		
Pentobarbital	225.0	42.4	53	48	3.78
		182.0	18		
IS	294.0	214.0	24	74	4.22
		249.9	14		
